# Four decades of ADHD: a systematic AI-assisted analysis of conceptual shifts across six DSM editions

**DOI:** 10.3389/fpsyt.2026.1792051

**Published:** 2026-03-09

**Authors:** Yaakov Ophir, Yaffa Shir-Raz, Refael Tikochinski

**Affiliations:** 1 Department of Education, Ariel University, Ariel, Israel; 2 Centre for Human-Inspired Artificial Intelligence (CHIA), University of Cambridge, Cambridge, United Kingdom; 3 Department of Communication, University of Haifa, Haifa, Israel; 4 Experimental Psychology Department, University College London (UCL), London, United Kingdom

**Keywords:** ADHD, AI-assisted text analysis, DSM, medicalization, psychiatric classification

## Abstract

**Background:**

Considering the central role of the *Diagnostic and Statistical Manual of Mental Disorders* (DSM) in psychiatric classification, multiple studies have examined how it describes *Attention-Deficit/Hyperactivity Disorder* (ADHD) – one of the most common psychiatric diagnoses. However, despite analyzing the same DSM texts, these studies yielded conflicting conclusions, likely influenced by the subjectivity of qualitative research and the challenge of systematically tracking subtle changes in large textual corpora. This study addresses these limitations by providing the first systematic, Artificial Intelligence (AI)-assisted analysis of all ADHD-related texts across six DSM editions (*DSM-III* to *DSM-5-TR*).

**Methods:**

The analysis employed two AI models (GPT-4o and Claude 3.5 Sonnet) and followed five structured steps: (A) preliminary human review, (B) AI-assisted comparative analysis, (C) refinement through AI self-prompting to detect subtle linguistic changes, such as tone and diagnostic uncertainties, (D) thematic synthesis by each model, and (E) cross-model validation. Strict adherence to DSM texts ensured all findings were grounded in verifiable textual evidence.

**Results:**

The analysis identified six overarching trends (1): a shift from a behavioral disorder to a neurodevelopmental framework (2), expansion to a lifespan condition across genders, (3) a broadening concept of impairment, (4) increasing diagnostic flexibility, (5) an expanding scope of comorbidities and differential diagnoses, and (6) growing acknowledgment of cultural and contextual influences.

**Conclusions:**

The six overarching shifts alongside the detailed systematic analysis results (**Supplementary Materials**) provide a transparent and replicable reference point for how ADHD has been described and classified in the DSM over four decades. Additionally, the innovative methodology can improve reliability of future research into complex psychiatric discourse.

## 1 Introduction

*Attention-Deficit/Hyperactivity Disorder* (ADHD)—one of the most common psychiatric diagnoses in children (1)—was introduced in the third edition of the *Diagnostic and Statistical Manual of Mental Disorders* (DSM-III; 2). Widely regarded as a turning point in psychiatry, DSM-III adopted a symptom-based, atheoretical approach that prioritized diagnostic reliability and standardization while aligning psychiatry with the medical model (3, 4). As part of this shift, it consolidated several earlier childhood classifications (e.g., Hyperkinetic Reaction of Childhood) into a single diagnosis—Attention Deficit Disorder—stating that “*attentional difficulties are prominent and virtually always present among children with these [earlier] diagnoses*” (2, p. 41).

Four decades and five revisions later, the current edition (DSM-5-TR; 5) retains a closely related label (Attention-Deficit/Hyperactivity Disorder) but reflects substantial conceptual shifts in how ADHD is defined, classified, and understood (6–9). These changes have been the focus of extensive research, not only because of the DSM’s authority as the leading psychiatric classification system but also due to its broader influence on clinical practice and societal discourse (10, 11).

On the one hand, studies supporting the DSM’s revisions typically interpret them as scientific advancements that enhance diagnostic accuracy. These studies often argue that the rising prevalence of ADHD (12–15) reflects improved recognition of previously underdiagnosed cases, particularly among adults and females (16–19). On the other hand, critics question the empirical basis of these changes and suggest that the increasing prevalence may reflect diagnostic expansion, raising concerns about potential overdiagnosis (20–22).

This divergence between supporters and critics—despite analyzing the same DSM texts—may reflect the inherent subjectivity and complexity of traditional text analysis, where conclusions are shaped less by quantifiable data and more by interpretive framing. Manual textual analysis makes it challenging to identify and compare subtle linguistic and structural changes across multiple DSM editions, and reviewers’ conclusions may be influenced by their theoretical or clinical orientations.

To date, all existing studies on how ADHD is portrayed in the DSM have relied on qualitative methods involving manual extraction of themes from long and complex texts (e.g., 16, 8). While such approaches offer valuable depth and nuance, they are limited in their ability to systematically and objectively track patterns over time. Moreover, to our knowledge, no prior study has conducted a comprehensive, cross-edition analysis of *all* DSM volumes that include ADHD, leaving an important gap in our understanding of how diagnostic definitions evolve.

## 2 The current study

**Figure 1:**
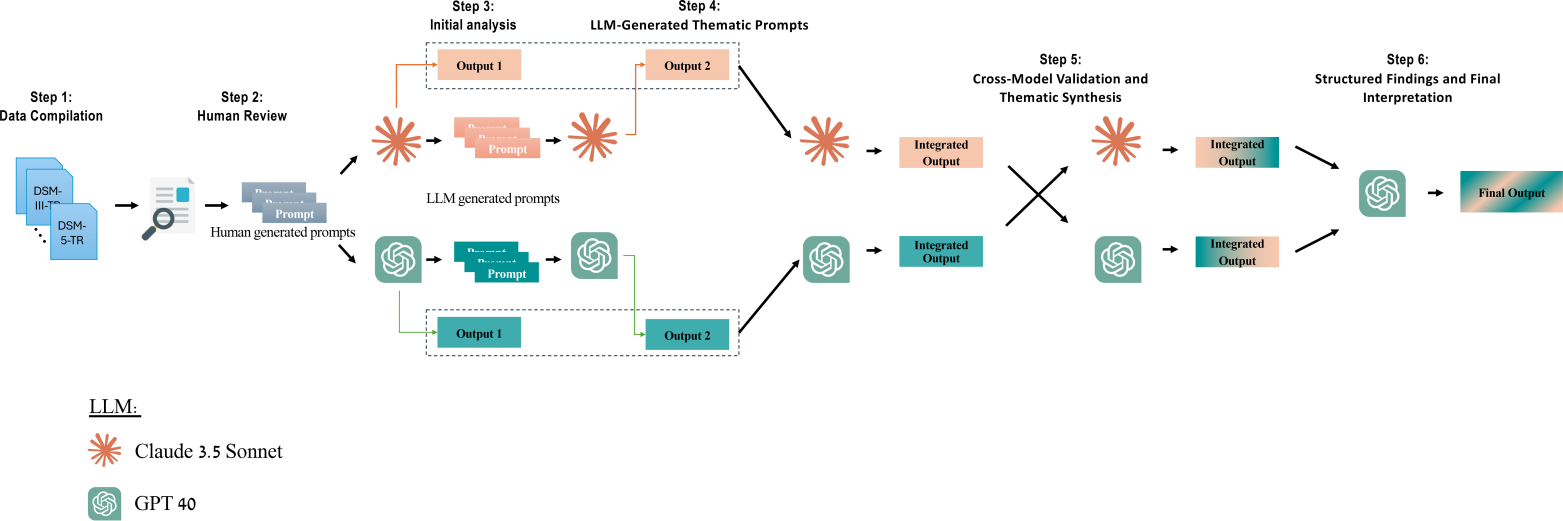
Overview of the five-stage AI-assisted analytical pipeline.

This study presents the first systematic analysis of all available descriptions of ADHD across the six editions of the psychiatric manual (DSM-III to DSM-5-TR). To address the limitations of prior research, we developed a structured analytical pipeline that leveraged two state-of-the-art AI language models (**Figure 1**). This approach enabled the identification of subtle textual changes across DSM editions, incorporated cross-validation between the models, and required all findings to be grounded in verbatim quotations from the DSM texts.

Importantly, we intentionally minimized potentially biased human interpretations throughout the process. All methodological steps, including the wording of prompts, model outputs, and validation procedures, are fully documented in the following sections and in the **Supplementary Materials**. In this way, the present study seeks to inform ongoing conceptual and clinical discussions about the nature and diagnostic boundaries of ADHD by establishing a transparent, evidence-based reference point. Rather than advancing a specific interpretive claim, our goal is to provide a structured foundation that can support future dialogue, critique, and conceptual clarification.

## 3 Methods

The dataset included all relevant ADHD-related texts from DSM-III (1980) through DSM-5-TR (2022), totaling 17,580 words. We analyzed this corpus using a structured five-step pipeline developed to systematically track conceptual and linguistic shifts in ADHD descriptions (**Figure 1**).

### 3.1 Step 1: preliminary human review

Two researchers manually examined the compiled DSM texts to familiarize themselves with key conceptual shifts and establish the first prompts for the subsequent AI-assisted analysis. This initial phase also provided a reference point for evaluating the accuracy, relevance, and consistency of the AI-generated results throughout the entire pipeline.

### 3.2 Step 2: initial AI-assisted comparative analysis

The full ADHD corpus was analyzed using two large language models, GPT-4o and Claude 3.5 Sonnet (23, 24), both of which have been increasingly discussed and applied in academic research for complex qualitative and comparative textual analysis tasks (25, 26). These models, developed independently by two different organizations, were used in parallel as a methodological precaution, enabling cross-model comparison and reducing reliance on any single system’s assumptions or idiosyncratic outputs.

In practice, each model was instructed to independently identify differences across DSM editions while remaining strictly bound to the original textual corpus. All AI-generated observations were required to be supported by verbatim quotations from the DSM texts and were subsequently verified against the original sources (**Supplementary Materials 1**, **2**).

### 3.3 Step 3: refinement of the analysis through AI self-prompting

To enhance the depth and precision of the analysis, each AI model was tasked with independently generating its own set of analytical prompts. This step was designed to reduce reliance on a predefined, human-authored analytical framework and to allow each model to operationalize the task according to its internal prompt-generation processes and response styles. By doing so, the analysis aimed to address limitations of traditional qualitative approaches, particularly in systematically detecting subtle textual changes across large and complex corpora. In this step, GPT-4o and Claude 3.5 Sonnet generated 10 and 7 targeted prompts, respectively, focusing on aspects such as shifts in language and tone, as well as diagnostic uncertainties (**Table 1**).

**Table 1:** Thematic focus of AI self-prompting in GPT-4o and Claude 3.5 sonnet.

GPT-4o	Claude 3.5
Conceptual evolution of ADHD over time Changes in diagnostic criteria Subtle shifts in descriptive language and tone Evolution of ADHD-related risk factors Changes in ADHD’s relationship with comorbid conditions Differences in functional impairment and prognosis descriptions Cultural and gender bias in ADHD diagnosis Neurobiological and genetic explanations over time Changes in ADHD prevalence estimates ADHD in adults: Emerging recognition	Language pattern analysis Contextual framework evolution Diagnostic boundary analysis Gender and cultural considerations Temporal language analysis Impairment conceptualization Diagnostic uncertainty

All model-generated prompts were manually reviewed to ensure their relevance and accuracy before being reintroduced into their respective models. Importantly, all prompts explicitly required the models to substantiate their findings with verbatim quotations from the DSM texts, which were attached as a document file each time (**Supplementary Materials 1**, **2**).

### 3.4 Step 4: thematic synthesis by each model

The complete AI-generated findings were then categorized into structured thematic groups by the respective AI models. Each model was instructed to: (a) summarize the most significant changes in each DSM edition, (b) identify overarching trends in how ADHD has been defined, framed, and classified over time, (c) analyze the implications of these changes, and (d) support all responses with verbatim quotations.

Altogether, this process (Steps 2–4) produced two comprehensive files: one containing the full analysis from GPT-4o (16,512 words) and another from Claude 3.5 Sonnet (8,631 words). These files, which represent the complete AI-assisted analyses of ADHD in the DSM, are available in **Supplementary Materials 1**, **2**.

### 3.5 Step 5: cross-model validation

In the final step, each model was provided with the two comprehensive files containing the full AI-assisted analyses and tasked with identifying key insights that both models independently recognized and agreed upon. The models were instructed to rely solely on the AI-generated analyses without introducing new interpretations or external knowledge (**Supplementary Material 3**).

Next, the two sets of agreed-upon insights were compiled and fed into GPT-4o, which was tasked with synthesizing a final summary of the changes that both models determined to be consensual. This final comparison represents the culmination of the methodological process, systematically consolidating the findings and highlighting the major conceptual shifts in ADHD across six DSM editions (**Supplementary Material 4**).

## 4 Results

The final results of the AI-assisted analytical pipeline (**Supplementary Material 4**) were manually reviewed, systematically organized, and synthesized into six overarching shifts.

### 4.1 Neurodevelopmental framework

In its initial formulation (DSM-III), ADHD was conceptualized primarily as a behavioral disorder. Over time, its classification evolved into a neurodevelopmental framework, incorporating genetic, neurobiological, and cognitive components (DSM-5, DSM-5-TR). DSM-5 also marked the first introduction of the distinct diagnostic category *Neurodevelopmental Disorders*, under which ADHD was placed. In all previous editions, ADHD had been classified under *Disorders Usually First Diagnosed in Infancy, Childhood, or Adolescence*.

Notably, despite this shift, recent editions (DSM-5 and DSM-5-TR) also mention that “*no biological marker is diagnostic for ADHD*.” DSM-5-TR further clarifies that “*meta-analyses of all neuroimaging studies do not show differences between individuals with ADHD and control subjects*” and concludes that “*until these issues are resolved, no form of neuroimaging can be used for diagnosis of ADHD*.”

### 4.2 Lifespan condition across genders

In its early editions, the DSM conceptualized ADHD as a childhood-specific disorder, with no formal recognition of persistence into adulthood (DSM-III, DSM-III-R). DSM-III estimated its prevalence at 3% among prepubertal children in the U.S, stating it was “*ten times more common in boys than in girls*.”

Over time, the reported prevalence among children steadily increased, reaching 7.2% in DSM-5-TR. Newer editions also acknowledged gender differences in ADHD presentation, with DSM-5-TR stating that “*females are more likely than males to present primarily with inattentive features*.” Correspondingly, the male-to-female prevalence ratio has gradually declined from 10:1 (DSM-III) to 6–9:1 (DSM-III-R), 4–9:1 (DSM-IV), and approximately 2:1 (DSM-5 and DSM-5-TR). Among adults the current reported ratio is 1.6:1.

In parallel, DSM-5 formally recognized adult ADHD, lowered the diagnostic threshold for individuals aged 17 and older, and acknowledged its persistence across the lifespan. The required age of onset was also extended from before age 7 (DSM-III through DSM-IV-TR) to before age 12 (DSM-5). DSM-5 also introduced the first specific prevalence estimate for adult ADHD (2.5%).

### 4.3 Impairment

Early DSM editions primarily defined ADHD based on core symptoms of inattention and hyperactivity, with impairment framed mostly in terms of academic underperformance (DSM-III). Over time, later editions (DSM-5, DSM-5-TR) placed increasing emphasis on functional impairment across multiple domains, including occupational, social, and emotional regulation difficulties.

A designated section on “*Functional Consequences of Attention-Deficit/Hyperactivity Disorder*” was introduced in DSM-5 and DSM-5-TR. DSM-5 listed severe negative consequences such as antisocial personality disorder, substance use disorders, incarceration, traffic accidents, obesity, and peer rejection. DSM-5-TR further extended this list, adding poor job stability, lower self-esteem, increased risk of trauma and subsequent PTSD, as well as a higher overall mortality rate—primarily due to accidents and injuries.

In addition to functional impairment, the relationship between ADHD and suicide risk was first introduced in DSM-5 and further elaborated in DSM-5-TR. DSM-5 mentioned an “*increased risk of suicide attempt*” under the section “*Associated Features Supporting Diagnosis.*” DSM-5-TR expanded on this by introducing a dedicated section titled “*Association With Suicidal Thoughts or Behavior*,” explicitly stating that “*ADHD is a risk factor for suicidal ideation and behavior in children.*”

Notably, however, the direct texts on the impairment criterion have fluctuated over the years in a non-linear trend:

- Early editions (DSM-III, DSM-III-R) set relatively mild dysfunction thresholds for diagnosis. DSM-III stated that “academic difficulties are common; and although impairment may be limited to academic functioning, social functioning may be impaired as well.” DSM-III-R similarly noted that “some impairment in social and school functioning is common” and introduced severity criteria, including a “mild” ADHD category, where symptoms caused “only minimal or no impairment in school and social functioning.”
- DSM-IV and DSM-IV-TR appear to have made the impairment criterion more stringent. The severity criteria were omitted, and a stricter “Criterion D” was introduced, requiring “clear evidence of clinically significant impairment in social, academic, or occupational functioning.”
- Finally, DSM-5 and DSM-5-TR reintroduced severity levels, once again allowing for a “mild” ADHD diagnosis. DSM-5-TR further introduced the category “in partial remission” to describe cases where individuals no longer meet the full diagnostic criteria but continue to experience some symptoms. Correspondingly, Criterion D was revised to require “clear evidence that the symptoms interfere with, or reduce the quality of, social, academic, or occupational functioning,” reflecting a more flexible approach to defining impairment.

### 4.4 Diagnostic flexibility

The current flexibility in the impairment criterion (Finding 3) appears to be part of a broader trend toward a more adaptable diagnostic framework. ADHD was initially categorized into subtypes, with DSM-III distinguishing between ADD with and without hyperactivity. DSM-IV restructured these classifications into three subtypes: Predominantly Inattentive, Predominantly Hyperactive-Impulsive, and Combined Type. DSM-5 later replaced subtypes with *presentations*, allowing for greater diagnostic flexibility and acknowledging that symptom profiles may change over time.

This shift is also reflected in the DSM’s evolving approach to subthreshold cases of ADHD.

- DSM-III (1980) introduced *Residual Type* of the disorder, which referred to individuals who had previously met full diagnostic criteria but continued to experience some symptoms in a reduced form.
- DSM-III-R (1987) removed Residual Type without a direct replacement but introduced a severity scale, including a “*mild*” ADHD category as mentioned above (Finding 3) – a category, which may have compensated for the removal of the Residual Type.
- DSM-IV and DSM-IV-TR replaced Residual Type with *ADHD Not Otherwise Specified (NOS)*, broadening the framework to include clinically significant cases that did not fully meet ADHD criteria, even if they had never met the full criteria before.
- DSM-5 and DSM-5-TR refined this structure further, replacing NOS with two distinct diagnostic alternatives: *Other Specified ADHD* (used when clinicians specify why a presentation does not fully meet ADHD criteria) and *Unspecified ADHD* (used when clinicians either do not specify the reason or lack sufficient information).

### 4.5 Comorbidities and differential diagnoses

In DSM-III, ADHD was primarily linked to conduct disorder and learning disabilities. Over time, DSM-5 and DSM-5-TR expanded its comorbidity profile to include autism spectrum disorder (ASD), mood disorders, substance use disorders, and anxiety disorders, reflecting a broader and more complex clinical framework (see also Finding 3).

Simultaneously, the differential diagnosis section expanded considerably. DSM-III and DSM-III-R listed 5 and 6 conditions, respectively, including “*age-appropriate overactivity*” as a diagnostic consideration. DSM-IV and DSM-IV-TR increased this to 10, while DSM-5 and DSM-5-TR further expanded it to 16 and 17. Notably, the updated lists excluded “*age-appropriate overactivity*” and did not include “*normal variations*,” a consideration found in the differential diagnosis of some other neurodevelopmental disorders.

### 4.6 Contextual and cultural considerations

Finally, despite the shift towards a neurodevelopmental framework (Finding 1), contemporary DSM editions have expanded their acknowledgment of environmental and contextual factors in shaping symptom expression. While earlier DSM editions made little to no reference to cultural considerations, DSM-5-TR explicitly recognizes racial and ethnic disparities, highlighting patterns of underdiagnosis in minority populations and the potential influence of clinician bias on diagnostic practices. Additionally, recent editions have incorporated environmental influences on symptom manifestation, acknowledging the roles of family dynamics, digital environments, and workplace settings in modulating the presentation and severity of ADHD symptoms.

## 5 Discussion

This study provides the first systematic, AI-assisted analysis of how ADHD has been described and classified in the DSM over the past four decades. By tracing changes across six DSM editions, the analysis identified six overarching trends:

1. ADHD has shifted from a primarily behavioral disorder to a neurodevelopmental condition, emphasizing biological and cognitive underpinnings.
2. Initially conceptualized as a disorder of young boys, ADHD has evolved into a lifespan condition affecting individuals across genders. The reported prevalence in children increased from 3% to 7.2%, while the male-to-female ratio declined from 10:1 to 2:1. Simultaneously, DSM-5 formally recognized adult ADHD (with a 2.5% prevalence), lowered the symptom threshold for older individuals, and extended the required age of onset from 7 to 12 years.
3. The definition of impairment has evolved, broadening beyond academic difficulties to include occupational, social, and emotional challenges. A range of negative consequences were introduced including, for example, incarceration, traffic accidents, overall mortality, and suicide. At the same time, the latest editions have also lowered the required threshold for impairment, allowing for the diagnosis of individuals with minor difficulties.
4. Diagnostic criteria have become more flexible, moving from rigid subtypes to a classification that accommodates changing symptom presentations, including cases that do not fully meet the diagnostic criteria (categorized as Other Specified and Unspecified ADHD).
5. The scope of ADHD’s comorbidities and differential diagnoses has expanded significantly, with DSM-5-TR listing 17 differential diagnoses—excluding ‘normal variations.’
6. While the DSM increasingly frames ADHD as a neurobiological disorder, later editions also acknowledge the role of cultural and environmental factors (e.g., digital environments) in shaping symptom expressions.

These findings were derived from a dual-model AI pipeline—based on GPT-4o and Claude 3.5 Sonnet—and subjected to cross-model validation (**Figure 1**). All findings were grounded in verbatim quotations from the DSM and detailed in four **Supplementary Materials**.

The contributions of this study are threefold. *First*, it leveraged the advanced language capabilities of large AI models to systematically detect subtle textual shifts across multiple and complex editions of the DSM. *Second*, it offers a relatively objective and reproducible alternative to traditional qualitative approaches, which—despite examining the same texts—have produced conflicting interpretations of ADHD’s evolving description (8, 16, 17, 20). *Third*, it provides a fully transparent account of its methods and findings (see the four **Supplementary Materials**), offering a comprehensive and replicable reference point for researchers, clinicians, and policymakers concerned with ADHD classification and its implications.

From a theoretical perspective, the findings of the present systematic and comparatively objective analysis may help illuminate several longstanding conceptual deliberations surrounding ADHD, including (A) the relationship between diagnostic expansion and observed prevalence rates (14, 18); (B) the implications of gender-related revisions for construct coherence (27); (C) the tension between neurodevelopmental framing and acknowledged contextual and social influences (15, 28–30); (D) the expanding scope of comorbidities and differential diagnoses (31, 32); and (E) the challenge of distinguishing pathological conditions from normative developmental variation (33, 34), which has important downstream implications for clinical decision-making, including considerations regarding treatment approaches (35, 36).

In the present study, we do not seek to resolve these complex issues. Rather, we aimed to present the analysis with minimal human interpretation, allowing readers to evaluate whether the observed conceptual shifts reflect improved identification of previously overlooked cases or a broadening of diagnostic boundaries that may carry a risk of overdiagnosis (37, 38). Accordingly, we suggest that future discussions consider these findings in light of the DSM’s own definition of a mental disorder, which emphasizes “clinically significant disturbance” and cautions against diagnosing “an expectable or culturally approved response to a common stressor” or “conflicts that are primarily between the individual and society” (5).

### 5.1 Limitations

While this study provides a well-validated analysis of ADHD-related texts, several limitations should be acknowledged. First, the study focused exclusively on DSM texts, which, while serving as the most authoritative psychiatric classification system, represent only one aspect of how ADHD is conceptualized. The evolution of ADHD is influenced by broader forces, including empirical research, clinical practice, educational policies, and public discourse. Second, while this study critically examined shifts in the DSM’s portrayal of ADHD, it did not empirically assess their impact on real-world clinical practice, such as changes in prevalence rates, prescribing patterns, or patient outcomes. Future research should integrate real-world data to examine how DSM modifications translate into clinical decision-making.

Third, while the analytical pipeline is fully transparent and can be re-applied to the same DSM corpus, the proprietary and continuously evolving nature of AI language models limits exact output-level reproducibility. Ongoing model updates may lead to variation in specific phrasing, although the analysis is designed to promote robustness at the level of quotation-grounded patterns rather than identical model-generated wording. Fourth, AI models are susceptible to biases based on their training data, including frequency effects that may skew their focus toward more recent DSM editions. Finally, AI models function as black-box systems, meaning their internal decision-making processes remain opaque (39). Although cross-validation measures were implemented and models were required to support all findings with verbatim DSM quotations to mitigate biases and hallucinations, human expertise remains essential for interpreting findings and contextualizing them within the broader scientific and clinical landscape.

Future studies may benefit from utilizing open-weight AI models, which make their parameters publicly available, improving transparency, reproducibility, and researcher control over the analysis. Additionally, integrating hybrid methodologies that combine AI analysis with traditional qualitative research may further strengthen findings by providing a more nuanced understanding of ADHD’s evolving conceptualization. Despite these limitations, this study establishes a structured, transparent, and replicable foundation for future research, offering valuable insights for researchers, clinicians, and policymakers seeking to better understand the shifting diagnostic landscape of ADHD.

## Data Availability

The original contributions presented in the study are included in the article/**Supplementary Material**. Further inquiries can be directed to the corresponding author.
