# Supplementary material for: Four decades of ADHD: a systematic AI-assisted analysis of conceptual shifts across six DSM editions
Source: Front Psychiatry. 2026 Mar 9;17:1792051. doi: 10.3389/fpsyt.2026.1792051 (PMC13006570; doi:10.3389/fpsyt.2026.1792051)
Supplement: Supplementary file 2 [file DataSheet2.pdf]

ADHD texts from DSM 3, 3R, 4, 4TR, 5, 5TR

DOCX

YO Attention-Deficit Disorder (ADD/ADHD) was first introduced in 1980 with the publication of the third edition of the Diagnostic and Statistical Manual of Mental Disorders (DSM-III). Since then, the DSM has undergone five revisions: DSM-III-R (1987), DSM-IV (1994), DSM-IV-TR (2000), DSM-5 (2013), and DSM-5-TR (2022).

Attached is a Word file containing the complete texts on ADD/ADHD from each edition of the DSM. Analyze these texts and identify the main differences between them.

For each difference you identify, provide exact verbatim quotations from the relevant DSM editions that explicitly support your observations. Clearly indicate which edition each excerpt is taken from.

I'll analyze the key changes in how ADHD has been conceptualized across DSM editions. Here are the major differences, supported by direct quotes:

1. Name and Classification Changes:

- DSM-III (1980) used "Attention Deficit Disorder" with subtypes "with Hyperactivity" and "without Hyperactivity"
- DSM-III-R (1987) changed to "Attention-deficit Hyperactivity Disorder (ADHD)" without subtypes
- DSM-IV (1994) and later editions used "Attention-Deficit/Hyperactivity Disorder" with new subtypes

Quotes:
DSM-III: "In this manual Attention Deficit is the name given to this disorder... There are two subtypes of the active disorder, Attention Deficit Disorder with Hyperactivity, and Attention Deficit Disorder without Hyperactivity"

DSM-III-R: "314.01 Attention-deficit Hyperactivity Disorder (ADHD)"

2. Age of Onset Criterion:

- DSM-III through DSM-IV-TR required symptoms before age 7
- DSM-5 and 5-TR changed this to age 12

Quotes:
DSM-IV: "Some hyperactive-impulsive or inattentive symptoms that caused impairment

were present before age 7 years"

DSM-5: "Several inattentive or hyperactive-impulsive symptoms were present prior to age 12 years"

3. Symptom Requirements for Adults:

- Earlier editions used same criteria for all ages
- DSM-5 introduced different thresholds for adults

Quote from DSM-5:
"For older adolescents and adults (age 17 and older), at least five symptoms are required" compared to six symptoms for children

4. Expanded Context Understanding:

- Later editions added more detail about how symptoms manifest in different settings and ages

Quote from DSM-5:
"Signs of the disorder may be minimal or absent when the person is receiving frequent rewards for appropriate behavior, is under close supervision, is in a novel setting, is engaged in especially interesting activities, has consistent external stimulation (e.g., via electronic screens), or is interacting in one-on-one situations"

5. Addition of Severity Specifiers:

- DSM-5 added formal severity levels

Quote from DSM-5:
"Specify current severity:
Mild: Few, if any, symptoms in excess of those required to make the diagnosis...
Moderate: Symptoms or functional impairment between 'mild' and 'severe'...
Severe: Many symptoms in excess of those required..."

6. Enhanced Understanding of Adult Presentation:

- Later editions added more detail about adult manifestations

Quote from DSM-5-TR:
"In adults, along with inattention and restlessness, impulsivity may remain problematic even when hyperactivity has diminished"

7. Cultural Considerations:

- DSM-5-TR added more extensive discussion of cultural factors

Quote from DSM-5-TR:
"Clinical identification rates in the United States for African American and Latinx populations tend to be lower than for non-Latinx White populations. Underdetection may result from mislabeling of ADHD symptoms as oppositional or disruptive in socially oppressed ethnic or racialized groups"

Would you like me to elaborate on any of these changes or explore additional differences between the editions?

YO What can be learned from the differences you identified about the discourse on ADHD throughout the years, since 1980 (DSM III) and 2022 (DSM-5-TR)?

The evolution of ADHD discourse across DSM editions reveals several important trends in how the psychiatric community's understanding and conceptualization of the disorder has developed:

1. Growing Recognition of Complexity and Heterogeneity:

- DSM-III's relatively simple model of presence/absence of hyperactivity evolved into a more nuanced understanding of multiple presentations
- The discourse shifted from viewing ADHD as primarily a childhood behavioral disorder to recognizing it as a complex neurodevelopmental condition that manifests differently across the lifespan
- This is reflected in the DSM-5-TR's extensive discussion of how symptoms present differently in various contexts and developmental stages

2. Increased Focus on Adult ADHD:

- Early editions (DSM-III/III-R) focused heavily on childhood presentations
- Later editions increasingly acknowledged and detailed adult manifestations
- This shift suggests growing recognition that ADHD is not just a childhood disorder but often persists into adulthood
- The adjustment of diagnostic criteria for adults (requiring fewer symptoms) in DSM-5 reflects better understanding of how the disorder evolves with age

3. Enhanced Understanding of Environmental Context:

- Earlier editions presented a more static view of symptoms
- Later editions increasingly emphasized how symptoms vary across different settings and situations
- This suggests a move toward a more ecological understanding of ADHD that considers how environmental factors influence symptom expression

4. Growing Social Justice Awareness:

- Early editions had minimal discussion of cultural factors
- DSM-5-TR explicitly addresses racial and ethnic disparities in diagnosis and treatment
- This reflects growing awareness of how social and cultural factors influence diagnosis and access to care

- The discussion of potential clinician bias and cultural differences in symptom interpretation shows increased attention to equity issues

5. Shift from Categorical to Dimensional Thinking:

- Early editions presented ADHD as more categorical (you either have it or don't)
- Later editions introduced severity specifiers and acknowledged partial remission
- This suggests movement toward viewing ADHD as existing on a spectrum rather than as a binary condition

6. Broadening of Age Criteria:

- The change in age of onset from 7 to 12 years reflects recognition that:
  - Symptoms may not become apparent until academic demands increase
  - Earlier strict criteria may have excluded individuals with valid diagnoses
- This shows a move toward more inclusive diagnostic criteria while maintaining the essential developmental nature of the disorder

7. Increasing Sophistication in Understanding Comorbidity:

- Later editions provide more detailed discussion of how ADHD interacts with other conditions
- This reflects growing recognition of ADHD's complexity and its relationships with other disorders
- The discourse has moved from viewing ADHD in isolation to understanding it as part of a potentially complex clinical picture

8. Evolution in Language and Terminology:

- The shift from "Attention Deficit Disorder" to "Attention-Deficit/Hyperactivity Disorder" reflects more precise clinical conceptualization
- Later editions use more nuanced and less stigmatizing language
- This suggests growing awareness of how diagnostic labels affect individuals and society

These changes reflect broader trends in psychiatry and society:

- Movement toward more nuanced, context-sensitive understanding of mental health conditions
- Growing awareness of social justice and equity issues
- Increasing sophistication in understanding neurodevelopmental conditions
- Recognition of the importance of lifespan development
- Greater attention to how disorders manifest in different populations and contexts

The evolution of ADHD discourse in the DSM mirrors broader societal changes in how we understand and approach neurodevelopmental differences, moving from a simpler, more categorical model to a more nuanced, contextual, and socially aware framework.

YO Some differences between the various editions of the DSM are relatively easy for humans to identify.

Suggest prompts that are particularly well-suited for a Large Language Model—ones that effectively leverage its ability to process extensive texts, extract nuanced insights, and detect subtle differences that may not be immediately apparent to human readers.

Ensure that each prompt explicitly states that a Word file containing the complete texts of the relevant sections from each DSM edition is attached. Additionally, include clear instructions to provide exact verbatim quotations from these texts to support the findings.

Here are some analytically sophisticated prompts that leverage an LLM's strengths for analyzing the DSM ADHD texts:

1. Language Pattern Analysis: "Analyze the attached Word file containing complete DSM texts on ADHD from DSM-III through DSM-5-TR. Identify shifts in linguistic patterns, including changes in:

- Certainty/uncertainty markers (e.g., 'may', 'often', 'typically')
- Causal language (e.g., 'due to', 'results from', 'leads to')
- Conditional statements (e.g., 'if...then' constructions) Provide exact quotations from each edition to demonstrate these patterns."

2. Contextual Framework Evolution: "Review the attached Word file containing ADHD texts from all DSM editions. Trace how the contextual framing of symptoms evolved by analyzing:

- References to different environments (home, school, work, social settings)
- Discussion of how symptoms manifest differently across contexts
- Changes in how environmental influences are described Support your analysis with verbatim quotes from each edition."

3. Diagnostic Boundary Analysis: "Using the attached Word file with complete DSM texts on ADHD, analyze how the boundaries between ADHD and other conditions have been defined across editions. Focus on:

- Changes in differential diagnosis sections
- Evolution of comorbidity discussions
- Shifts in how overlapping symptoms are attributed Include exact quotations to demonstrate these changes."

4. Gender and Cultural Considerations: "Examine the attached Word file containing all DSM editions' ADHD texts. Analyze how discussions of gender and cultural factors

have evolved, including:

- Changes in terminology and framing
- Expansion or modification of considerations
- Shifts in how differences are explained Provide verbatim quotes to support your findings."

5. Temporal Language Analysis: "Using the attached Word file with complete DSM ADHD texts, analyze how temporal aspects are discussed across editions, including:

- Duration requirements
- Age-related specifications
- Development and course descriptions Include exact quotations demonstrating these temporal elements."

6. Impairment Conceptualization: "Review the attached Word file containing ADHD texts from all DSM editions. Analyze how the concept of impairment has evolved, focusing on:

- Changes in how functional impact is defined
- Evolution of severity descriptions
- Shifts in how impairment is measured or assessed Support your analysis with verbatim quotes from each edition."

7. Diagnostic Uncertainty: "Using the attached Word file with complete DSM texts, analyze how diagnostic uncertainty is addressed across editions by examining:

- Language about diagnostic challenges
- Discussion of borderline cases
- Evolution of 'Not Otherwise Specified' categories Include exact quotations demonstrating these elements."

These prompts are particularly suitable for LLMs because they:

- Require processing and comparing large amounts of text simultaneously
- Focus on subtle linguistic patterns and changes
- Need systematic comparison across multiple documents
- Benefit from the ability to detect minor variations in terminology and framing
- Require understanding context while maintaining attention to detail
- Can leverage the model's ability to identify patterns across different hierarchical levels
- Take advantage of the model's capacity to track complex changes across multiple dimensions simultaneously

Would you like me to elaborate on any of these prompts or suggest additional approaches?

ADHD texts from DSM 3, 3R, 4, 4TR, 5, 5TR

DOCX

YO Analyze the attached Word file containing complete DSM texts on ADHD from DSM-III through DSM-5-TR. Identify shifts in linguistic patterns, including changes in:
* Certainty/uncertainty markers (e.g., 'may', 'often', 'typically')
* Causal language (e.g., 'due to', 'results from', 'leads to')
Conditional statements (e.g., 'if...then' constructions) Provide exact quotations from each edition to demonstrate these patterns.

I'll analyze the linguistic patterns across DSM editions related to certainty/uncertainty and causation in their discussion of ADHD. Here are the key patterns I observe:

1. Evolution of Certainty Language:

DSM-III (1980) used more definitive language:

- "The essential features are signs of developmentally inappropriate inattention and impulsivity"
- "Onset is typically by the age of three"

By DSM-5/5-TR, language became more nuanced with increased use of qualifiers:

- "These traits may predispose some children to ADHD but are not specific to the disorder"
- "Symptoms typically vary depending on context"

2. Shift in Causal Language:

DSM-III used simpler causal constructions:

- "due to the child's psychopathology"
- "not due to cognitive impairment"

Later editions show more complex multifactorial causation:
DSM-5-TR:

- "Family interaction patterns in early childhood are unlikely to cause ADHD but may influence its course"
- "Neurotoxin exposure... has been correlated with subsequent ADHD, but it is not known whether these associations are causal"

3. Increasing Use of Uncertainty Markers:

DSM-III frequently used direct statements:

- "The disorder is apparently more common in family members"

DSM-5/5-TR shows more hedging:

- "Some personality disorders tend to share features..."
- "Visual and hearing impairments...should be considered as possible influences"

4. Evolution of Conditional Language:

Early editions used simpler if-then constructions:
DSM-III: "When the reports of teachers and parents conflict, primary consideration should be given to the teacher reports"

Later editions show more complex conditional relationships:
DSM-5-TR: "If clinically significant symptoms remain but criteria are no longer met for any of the subtypes, the appropriate diagnosis is Attention-Deficit/Hyperactivity Disorder, In Partial Remission"

This linguistic evolution reflects:

- Growing recognition of ADHD's complexity
- Increased emphasis on contextual factors
- More nuanced understanding of causation
- Greater acknowledgment of diagnostic uncertainty

Would you like me to provide additional examples or analyze any specific aspect in more detail?

ADHD texts from DSM 3, 3R, 4, 4TR, 5, 5TR

DOCX

YO Review the attached Word file containing ADHD texts from all DSM editions. Trace how the contextual framing of symptoms evolved by analyzing:
* References to different environments (home, school, work, social settings)
* Discussion of how symptoms manifest differently across contexts
Changes in how environmental influences are described Support your analysis with verbatim quotes from each edition.

I'll analyze how the DSM's treatment of context and environment in ADHD has evolved across editions, focusing on key changes in how symptoms are understood across different settings.

1. Evolution from Simple to Complex Environmental Framing:

DSM-III (1980) had a relatively simple environmental framing:
"A child's behavior may be well-organized and appropriate on a one-to-one basis but become dysregulated in a group situation or in the classroom; or home adjustment may be satisfactory and difficulties may emerge only in school."

By DSM-5-TR (2022), the framing became more nuanced:
"Signs of the disorder may be minimal or absent when the individual is receiving frequent rewards for appropriate behavior, is under close supervision, is in a novel setting, is engaged in especially interesting activities, has consistent external stimulation (e.g., via electronic screens), or is interacting in one-on-one situations."

2. Expansion of Environmental Contexts:

Early editions focused primarily on home and school:
DSM-III: "In the classroom... At home, attentional problems are shown by a failure to follow through on parental requests"

Later editions expanded to include:

- Work environments
- Social situations
- Recreational settings
- Digital contexts (e.g., electronic screens)

3. Growing Recognition of Contextual Variability:

DSM-5/5-TR introduced more sophisticated understanding of symptom variation:
"Typically, symptoms vary depending on context within a given setting... Confirmation of substantial symptoms across settings typically cannot be done accurately without consulting informants who have seen the individual in those settings."

4. Environmental Influences on Diagnosis:

Development in understanding cultural and socioeconomic factors:
DSM-5-TR acknowledges: "Prevalence is also affected by cultural variation in attitudes toward behavioral norms and expectations of children and youth in different social contexts"

5. Evolution in Understanding Environmental Modifiers:

Early editions saw environment mostly as potential causes:
DSM-III discussed "inadequate, disorganized, or chaotic environments"

Later editions shifted to seeing environment as a modifier:
DSM-5-TR states: "Family interaction patterns in early childhood are unlikely to cause ADHD but may influence its course"

This evolution shows increasing sophistication in understanding how ADHD manifests across different contexts and how environmental factors influence its expression and

assessment.

Would you like me to expand on any of these aspects?

ADHD texts from DSM 3, 3R, 4, 4TR, 5, 5TR

DOCX

YO Using the attached Word file with complete DSM texts on ADHD, analyze how the boundaries between ADHD and other conditions have been defined across editions.
Focus on:
* Changes in differential diagnosis sections
* Evolution of comorbidity discussions
Shifts in how overlapping symptoms are attributed Include exact quotations to demonstrate these changes.

I'll analyze how the boundaries between ADHD and other conditions evolved across DSM editions, focusing on key shifts in differential diagnosis and comorbidity.

1. Evolution of Differential Diagnosis Sophistication:

DSM-III (1980) had relatively simple differential diagnoses focused mainly on:

- Mental Retardation
- Conduct Disorder
- Schizophrenia and Affective Disorders

Quote: "In Schizophrenia and Affective Disorders with manic features there may be clinical features that are characteristic of Attention Deficit Disorder. However, these diagnoses preempt the diagnosis of Attention Deficit Disorder."

By DSM-5-TR (2022), the differential diagnosis section became much more nuanced, including:

- Detailed comparisons with multiple conditions
- Specific guidance on distinguishing overlapping symptoms
- Recognition of possible comorbidity

Quote: "Individuals with ADHD and those with autism spectrum disorder exhibit inattention, social dysfunction, and difficult-to-manage behavior. The social dysfunction and peer rejection seen in individuals with ADHD must be distinguished from the social disengagement, isolation, and indifference to facial and tonal communication cues seen in individuals with autism spectrum disorder."

2. Comorbidity Understanding:

DSM-III treated comorbidity minimally, mainly noting "associated features."

DSM-IV introduced more extensive comorbidity discussions:
Quote: "A substantial proportion of children with Attention-Deficit/Hyperactivity Disorder also have Oppositional Defiant Disorder or Conduct Disorder. There may be a higher prevalence of Mood Disorders, Anxiety Disorders, Learning Disorders, and Communication Disorders."

DSM-5/5-TR expanded this significantly:
Quote: "Although ADHD is more common in males, females with ADHD have higher rates of a number of comorbid disorders, particularly oppositional defiant disorder, autism spectrum disorder, and personality and substance use disorders."

3. Key Changes in Symptom Attribution:

Early editions (DSM-III/III-R) tended to view overlapping symptoms as either/or situations.

Later editions recognized more complex relationships:
Quote from DSM-5-TR: "Most children and adolescents with disruptive mood dysregulation disorder have symptoms that also meet criteria for ADHD; a lesser percentage of children with ADHD have symptoms that meet criteria for disruptive mood dysregulation disorder."

4. Evolution in Understanding Secondary Features:

DSM-III-R viewed secondary problems simply:
Quote: "Academic underachievement is characteristic of most children with this disorder."

DSM-5-TR provides more nuanced understanding:
Quote: "Inadequate or variable self-application to tasks that require sustained effort is often interpreted by others as laziness, irresponsibility, or failure to cooperate."

5. Recognition of Cultural and Contextual Factors:

DSM-5-TR added important considerations about diagnostic boundaries:
Quote: "Underdetection may result from mislabeling of ADHD symptoms as oppositional or disruptive in socially oppressed ethnic or racialized groups because of explicit or implicit clinician bias, leading to overdiagnosis of disruptive disorders."

This evolution shows increasing sophistication in understanding how ADHD relates to and interacts with other conditions, moving from simple exclusionary criteria to complex, nuanced relationships that recognize both distinct and overlapping features.

Would you like me to elaborate on any of these aspects or explore additional changes in diagnostic boundaries?

ADHD texts from DSM 3, 3R, 4, 4TR, 5, 5TR

DOCX

YO Examine the attached Word file containing all DSM editions' ADHD texts. Analyze how discussions of gender and cultural factors have evolved, including:
* Changes in terminology and framing
* Expansion or modification of considerations
Shifts in how differences are explained Provide verbatim quotes to support your findings.

I'll analyze how gender and cultural considerations in ADHD have evolved across DSM editions, with direct quotes showing key changes.

1. Gender/Sex Considerations:

DSM-III (1980) had minimal discussion, with just a basic ratio:
Quote: "The disorder is ten times more common in boys than in girls."

DSM-IV (1994) expanded this but still focused mainly on ratios:
Quote: "The disorder is much more frequent in males than in females, with male-to-female ratios ranging from 4:1 to 9:1, depending on the setting (i.e., general population or clinics)."

DSM-5-TR (2022) provides more nuanced understanding:
Quote: "ADHD is more frequent in males than in females in the general population, with a ratio of approximately 2:1 in children and 1.6:1 in adults. Females are more likely than males to present primarily with inattentive features. Sex differences in ADHD symptom severity may be due to differing genetic and cognitive liabilities between sexes."

2. Clinical Presentation by Gender:

DSM-5/5-TR introduced important distinctions in comorbidity patterns:
Quote: "Although ADHD is more common in males, females with ADHD have higher rates of a number of comorbid disorders, particularly oppositional defiant disorder, autism spectrum disorder, and personality and substance use disorders."

3. Cultural Considerations Evolution:

DSM-III had no specific cultural considerations.

DSM-IV (1994) introduced basic cultural acknowledgment:
Quote: "Attention-Deficit/Hyperactivity Disorder is known to occur in various cultures, with variations in reported prevalence among Western countries probably arising more from different diagnostic practices than from differences in clinical presentation."

DSM-5-TR (2022) provides comprehensive cultural analysis:
Quote: "Differences in ADHD prevalence across regions appear attributable mainly to different diagnostic procedures and methodological practices... Prevalence is also affected by cultural variation in attitudes toward behavioral norms and expectations of children and youth in different social contexts, as well as cultural differences in interpretations of children's behaviors by parents and teachers."

4. Recognition of Disparities:

DSM-5-TR introduced discussion of racial/ethnic disparities:
Quote: "Clinical identification rates in the United States for African American and Latinx populations tend to be lower than for non-Latinx White populations. Underdetection may result from mislabeling of ADHD symptoms as oppositional or disruptive in socially oppressed ethnic or racialized groups because of explicit or implicit clinician bias, leading to overdiagnosis of disruptive disorders."

5. Cultural Competence in Assessment:

DSM-5-TR emphasizes culturally competent practice:
Quote: "Informant symptom ratings may be influenced by the cultural background of the child and the informant, suggesting that culturally competent diagnostic practices are relevant in assessing ADHD."

This evolution shows:

- Movement from simple demographic ratios to nuanced understanding of gender differences
- Growing recognition of cultural influences on diagnosis and presentation
- Increased attention to disparities in diagnosis and treatment
- Greater emphasis on cultural competence in assessment
- Recognition of how social and cultural factors influence symptom interpretation

Would you like me to elaborate on any of these aspects?

YO Previously, in a separate chat window, I asked you to analyze how temporal aspects are discussed across DSM editions, specifically examining:

- Duration requirements
- Age-related specifications
- Development and course descriptions

Below is the exact response you provided in that chat. Please copy and paste it verbatim into the current chat window, without any modifications, summarization, or rewording.

"I'll analyze how temporal aspects of ADHD are discussed across DSM editions, focusing on duration requirements, age specifications, and developmental course descriptions. Let me organize this systematically. **Temporal Analysis of ADHD Across DSM Editions**

Duration Requirements

DSM-III (1980)

* Requires "Duration of at least six months" for diagnosis
* Applies this duration requirement to all symptoms listed under inattention, impulsivity, and hyperactivity

DSM-III-R (1987)

* Maintains six-month duration requirement: "symptoms have persisted for at least 6 months"
* Applies to both inattention and hyperactivity-impulsivity symptoms

DSM-IV (1994) & DSM-IV-TR (2000)

* Continues six-month requirement: "symptoms of inattention and/or hyperactivity-impulsivity have persisted for at least 6 months"
* Specifies symptoms must be "maladaptive and inconsistent with developmental level"

DSM-5 (2013) & DSM-5-TR (2022)

* Maintains six-month duration requirement for symptoms
* Specifies "persistent pattern" that "interferes with functioning or development"**Age-Related Specifications**

DSM-III (1980)

* Requires "Onset before the age of seven"
* Notes that onset is "typically by the age of three" Quote: "Onset is typically by the age of three, although frequently the disorder does not come to professional attention until the child enters school."

DSM-III-R (1987)

Quote: "In approximately half of the cases, onset of the disorder is before age four. Frequently the disorder is not recognized until the child enters school."

DSM-IV (1994) & DSM-IV-TR (2000)

* Maintains requirement that "symptoms that cause impairment were present before age 7 years"
* Acknowledges that "many individuals are diagnosed after the symptoms have been present for a number of years"

DSM-5 (2013) & DSM-5-TR (2022)

* Changes age requirement to "several inattentive or hyperactive-impulsive symptoms were present prior to age 12 years"
* More explicit about diagnostic challenges in early childhood Quote: "It is difficult to establish this diagnosis in children younger than age 4 or 5 years, because their characteristic behavior is much more variable than that of older children"**Development and Course**

DSM-III (1980)

Identifies three characteristic courses:

1. "All of the symptoms persist into adolescence or adult life"
2. "The disorder is self-limited and all of the symptoms disappear completely at puberty"
3. "The hyperactivity disappears, but the attentional difficulties and impulsivity persist"

DSM-III-R (1987)

Quote: "In the majority of cases manifestations of the disorder persist throughout childhood. Follow-up studies of clinic samples indicate that approximately one-third of children with ADHD continue to show some signs of the disorder in adulthood."

DSM-IV (1994) & DSM-IV-TR (2000)

More detailed developmental progression: Quote: "In most individuals, symptoms attenuate during late adolescence and adulthood, although a minority experience the full complement of symptoms into mid-adulthood."

DSM-5 (2013) & DSM-5-TR (2022)

Most comprehensive developmental description: Quote: "The disorder is relatively stable through early adolescence, but some individuals have a worsened course with development of antisocial behaviors. In most individuals with ADHD, symptoms of motoric hyperactivity become less obvious in adolescence and adulthood, but difficulties with restlessness, inattention, poor planning, and impulsivity persist."**Key Temporal Changes Across Editions**

1. **Age of Onset**
   * Shifted from age 7 (DSM-III through DSM-IV-TR) to age 12 (DSM-5/5-TR)
   * Recognition of diagnostic challenges in very young children became more explicit
1. **Duration Requirements**
   * Consistently maintained 6-month requirement across all editions
   * Language evolved to emphasize "persistent pattern" rather than just symptom presence
1. **Developmental Course**
   * Earlier editions (DSM-III/III-R) presented more categorical outcomes
   * Later editions provide more nuanced description of symptom evolution
   * Increasing recognition of adult ADHD and varying developmental trajectories

This analysis shows a clear evolution in how temporal aspects of ADHD are conceptualized across DSM editions. The most significant changes include:

1. A shift from rigid age-of-onset requirements to more flexible criteria that better reflect clinical reality

reflect clinical reality

2. Increasingly sophisticated understanding of how symptoms manifest differently across development
3. Greater recognition of ADHD as a potentially lifelong condition rather than primarily a childhood disorder."

Here is my previous response copied verbatim:

I'll analyze how temporal aspects of ADHD are discussed across DSM editions, focusing on duration requirements, age specifications, and developmental course descriptions. Let me organize this systematically.

Temporal Analysis of ADHD Across DSM Editions

Duration Requirements

DSM-III (1980)

- Requires "Duration of at least six months" for diagnosis
- Applies this duration requirement to all symptoms listed under inattention, impulsivity, and hyperactivity

DSM-III-R (1987)

- Maintains six-month duration requirement: "symptoms have persisted for at least 6 months"
- Applies to both inattention and hyperactivity-impulsivity symptoms

DSM-IV (1994) & DSM-IV-TR (2000)

- Continues six-month requirement: "symptoms of inattention and/or hyperactivity-impulsivity have persisted for at least 6 months"
- Specifies symptoms must be "maladaptive and inconsistent with developmental level"

DSM-5 (2013) & DSM-5-TR (2022)

- Maintains six-month duration requirement for symptoms
- Specifies "persistent pattern" that "interferes with functioning or development"

Age-Related Specifications

DSM-III (1980)

- Requires "Onset before the age of seven"
- Notes that onset is "typically by the age of three" Quote: "Onset is typically by the age of three, although frequently the disorder does not come to professional attention until the child enters school."

DSM-III-R (1987)
Quote: "In approximately half of the cases, onset of the disorder is before age four. Frequently the disorder is not recognized until the child enters school."

DSM-IV (1994) & DSM-IV-TR (2000)

- Maintains requirement that "symptoms that cause impairment were present before age 7 years"
- Acknowledges that "many individuals are diagnosed after the symptoms have been present for a number of years"

DSM-5 (2013) & DSM-5-TR (2022)

- Changes age requirement to "several inattentive or hyperactive-impulsive symptoms were present prior to age 12 years"
- More explicit about diagnostic challenges in early childhood Quote: "It is difficult to establish this diagnosis in children younger than age 4 or 5 years, because their characteristic behavior is much more variable than that of older children"

Development and Course

DSM-III (1980)
Identifies three characteristic courses:

1. "All of the symptoms persist into adolescence or adult life"
2. "The disorder is self-limited and all of the symptoms disappear completely at puberty"
3. "The hyperactivity disappears, but the attentional difficulties and impulsivity persist"

DSM-III-R (1987)
Quote: "In the majority of cases manifestations of the disorder persist throughout childhood. Follow-up studies of clinic samples indicate that approximately one-third of children with ADHD continue to show some signs of the disorder in adulthood."

DSM-IV (1994) & DSM-IV-TR (2000)
More detailed developmental progression:
Quote: "In most individuals, symptoms attenuate during late adolescence and adulthood, although a minority experience the full complement of symptoms into mid-adulthood."

DSM-5 (2013) & DSM-5-TR (2022)
Most comprehensive developmental description:
Quote: "The disorder is relatively stable through early adolescence, but some individuals have a worsened course with development of antisocial behaviors. In most individuals with ADHD, symptoms of motoric hyperactivity become less obvious in adolescence and adulthood, but difficulties with restlessness, inattention, poor planning, and impulsivity persist."

Key Temporal Changes Across Editions

1. **Age of Onset**

- Shifted from age 7 (DSM-III through DSM-IV-TR) to age 12 (DSM-5/5-TR)
- Recognition of diagnostic challenges in very young children became more explicit

2. **Duration Requirements**

- Consistently maintained 6-month requirement across all editions
- Language evolved to emphasize "persistent pattern" rather than just symptom presence

3. **Developmental Course**

- Earlier editions (DSM-III/III-R) presented more categorical outcomes
- Later editions provide more nuanced description of symptom evolution
- Increasing recognition of adult ADHD and varying developmental trajectories

This analysis shows a clear evolution in how temporal aspects of ADHD are conceptualized across DSM editions. The most significant changes include:

1. A shift from rigid age-of-onset requirements to more flexible criteria that better reflect clinical reality
2. Increasingly sophisticated understanding of how symptoms manifest differently across development
3. Greater recognition of ADHD as a potentially lifelong condition rather than primarily a childhood disorder.

YO Previously, in a separate chat window, I asked you to analyze how the concept of impairment has evolved, focusing on:

· Changes in how functional impact is defined
· Evolution of severity descriptions
· Shifts in how impairment is measured or assessed

Below is the exact response you provided in that chat. Please copy and paste it **verbatim** into the current chat window, without any modifications, summarization, or rewording.

"I'll analyze how the concept of impairment in ADHD has evolved across DSM editions, using quotes from the provided document.

Evolution of Impairment Concepts in ADHD Across DSM Editions
DSM-III (1980)
Functional Impact Definition
* Primarily focused on academic and social domains
* Quote: "Academic difficulties are common; and although impairment may be limited to academic functioning, social functioning may be impaired as well."
* Minimal discussion of occupational impact
Severity Description
* Limited severity criteria
* Focused on need for intervention: "Infrequently children with this disorder require residential treatment."
DSM-III-R (1987)
Functional Impact Definition
* Broadened to include multiple contexts
* Quote: "Some impairment in social and school functioning is common"
Severity Categories
Introduced formal severity levels:
* "Mild: Few, if any, symptoms in excess of those required to make the diagnosis and only minimal or no impairment in school and social functioning"
* "Moderate: Symptoms or functional impairment intermediate between 'mild' and 'severe'"
* "Severe: Many symptoms in excess of those required to make the diagnosis and significant and pervasive impairment in functioning at home and school and with peers"**DSM-IV (1994) & DSM-IV-TR (2000)**
Functional Impact Definition
* More comprehensive approach to impairment
* Required "clear evidence of clinically significant impairment in social, academic, or occupational functioning"
* Added context specificity: "To make the diagnosis, some impairment must be

present in at least two settings"

Contextual Considerations

Quote: "Symptoms typically worsen in situations that require sustained attention or mental effort or that lack intrinsic appeal or novelty"

DSM-5 (2013) & DSM-5-TR (2022)

Functional Impact Definition

Most comprehensive framework:

* Emphasizes interference with "functioning or development"
* Requires symptoms that "negatively impacts directly on social and academic/occupational activities"

Severity Specifications

Refined severity criteria:

1. Mild: "Symptoms result in no more than minor impairments in social or occupational functioning"
2. Moderate: "Symptoms or functional impairment between 'mild' and 'severe'"
3. Severe: "Symptoms result in marked impairment in social or occupational functioning"

Notable Additions

* Added specific language about adult impairment
* Included broader range of functional impacts: Quote: "Even in the absence of a specific learning disorder, academic or work performance is often impaired"

Key Evolution Points

1. Expansion of Domains
   * From primarily academic focus in DSM-III
   * To comprehensive life-domain impact in DSM-5/5-TR
2. Assessment Approach
   * Early editions: General descriptions of impairment
   * Later editions: Specific criteria for different settings and severity levels
3. Developmental Perspective
   * Increased recognition of age-specific impairments
   * Greater attention to adult manifestations
4. Contextual Considerations
   * Growing emphasis on situational variability
   * Recognition of environmental influences on impairment

This analysis reveals several key trends in how impairment has been conceptualized over time:

1. Increasing Sophistication: The understanding of impairment has become more nuanced, moving from simple academic/social difficulties to a complex interplay of functional domains.
2. Greater Specificity: Later editions provide more detailed criteria for assessing impairment, making diagnosis more standardized and reliable.
3. Lifespan Perspective: There's been a shift from child-focused impairment to consideration of how ADHD affects individuals across their lifespan.

4. Contextual Understanding: Recent editions emphasize how impairment varies across settings and situations, reflecting a more sophisticated understanding of the disorder."

Here is my previous response copied verbatim:

I'll analyze how the concept of impairment in ADHD has evolved across DSM editions, using quotes from the provided document.

Evolution of Impairment Concepts in ADHD Across DSM Editions

DSM-III (1980)
Functional Impact Definition

- Primarily focused on academic and social domains
- Quote: "Academic difficulties are common; and although impairment may be limited to academic functioning, social functioning may be impaired as well."
- Minimal discussion of occupational impact

Severity Description

- Limited severity criteria
- Focused on need for intervention: "Infrequently children with this disorder require residential treatment."

DSM-III-R (1987)
Functional Impact Definition

- Broadened to include multiple contexts
- Quote: "Some impairment in social and school functioning is common"

Severity Categories
Introduced formal severity levels:

- "Mild: Few, if any, symptoms in excess of those required to make the diagnosis and only minimal or no impairment in school and social functioning"
- "Moderate: Symptoms or functional impairment intermediate between 'mild' and 'severe'"
- "Severe: Many symptoms in excess of those required to make the diagnosis and significant and pervasive impairment in functioning at home and school and with peers"

DSM-IV (1994) & DSM-IV-TR (2000)
Functional Impact Definition

- More comprehensive approach to impairment
- Required "clear evidence of clinically significant impairment in social, academic, or occupational functioning"

- Added context specificity: "To make the diagnosis, some impairment must be present in at least two settings"

Contextual Considerations
Quote: "Symptoms typically worsen in situations that require sustained attention or mental effort or that lack intrinsic appeal or novelty"

DSM-5 (2013) & DSM-5-TR (2022)
Functional Impact Definition
Most comprehensive framework:

- Emphasizes interference with "functioning or development"
- Requires symptoms that "negatively impacts directly on social and academic/occupational activities"

Severity Specifications
Refined severity criteria:

1. Mild: "Symptoms result in no more than minor impairments in social or occupational functioning"
2. Moderate: "Symptoms or functional impairment between 'mild' and 'severe'"
3. Severe: "Symptoms result in marked impairment in social or occupational functioning"

Notable Additions

- Added specific language about adult impairment
- Included broader range of functional impacts: Quote: "Even in the absence of a specific learning disorder, academic or work performance is often impaired"

Key Evolution Points

1. Expansion of Domains

- From primarily academic focus in DSM-III
- To comprehensive life-domain impact in DSM-5/5-TR

2. Assessment Approach

- Early editions: General descriptions of impairment
- Later editions: Specific criteria for different settings and severity levels

3. Developmental Perspective

- Increased recognition of age-specific impairments
- Greater attention to adult manifestations

4. Contextual Considerations

- Growing emphasis on situational variability
- Recognition of environmental influences on impairment

This analysis reveals several key trends in how impairment has been conceptualized over time:

1. Increasing Sophistication: The understanding of impairment has become more nuanced, moving from simple academic/social difficulties to a complex interplay of functional domains.
2. Greater Specificity: Later editions provide more detailed criteria for assessing impairment, making diagnosis more standardized and reliable.
3. Lifespan Perspective: There's been a shift from child-focused impairment to consideration of how ADHD affects individuals across their lifespan.
4. Contextual Understanding: Recent editions emphasize how impairment varies across settings and situations, reflecting a more sophisticated understanding of the disorder.

YO Previously, in a separate chat window, I asked you to analyze how diagnostic uncertainty is addressed across editions by examining:
* Language about diagnostic challenges
* Discussion of borderline cases
* Evolution of 'Not Otherwise Specified' categories Include exact quotations demonstrating these elements."

Below is the exact response you provided in that chat. Please copy and paste it **verbatim** into the current chat window, without any modifications, summarization, or rewording.

"I'll analyze how the DSM editions have handled diagnostic uncertainty in ADHD. Let me organize this systematically.

Analysis of Diagnostic Uncertainty in ADHD Across DSM Editions

DSM-III (1980)
Diagnostic Challenges
* Acknowledged fundamental uncertainty about disorder subtypes Quote: "It is not known whether they are two forms of a single disorder or represent two distinct disorders"
Borderline Cases
* Highlighted difficulty distinguishing from environmental factors Quote: "In such cases it may be impossible to determine whether the disorganized behavior is simply a function of the chaotic environment or whether it is due to the child's psychopathology"
Residual Category
* Introduced "Residual Type" for partial presentations
* No explicit "Not Otherwise Specified" category

DSM-III-R (1987)
Diagnostic Challenges
* Added specificity about symptom variability Quote: "Signs of the disorder may be minimal or absent when the person is receiving frequent reinforcement or very strict control"
Borderline Cases
* More detailed discussion of differential diagnosis, especially with mood disorders
* Emphasized careful consideration in distinguishing from age-appropriate behavior
Diagnostic Uncertainty
* Required symptoms to be "considerably more frequent than that of most people of the same mental age"

DSM-IV (1994) & DSM-IV-TR (2000)

Diagnostic Challenges

* Expanded discussion of age-related diagnostic difficulties Quote: "It is especially difficult to establish this diagnosis in children younger than age 4 or 5 years, because their characteristic behavior is much more variable"

Diagnostic Uncertainty Language

* Introduced "In Partial Remission" category
* Added "Not Otherwise Specified" (NOS) category with specific examples

NOS Category Definition

Quote: "This category is for disorders with prominent symptoms of inattention or hyperactivity-impulsivity that do not meet criteria for Attention-Deficit/Hyperactivity Disorder"

DSM-5 (2013) & DSM-5-TR (2022)

Diagnostic Challenges

1. More explicit about recall reliability issues Quote: "Adult recall of childhood symptoms tends to be unreliable, and it is beneficial to obtain ancillary information"
2. Age-related uncertainty Quote: "ADHD cannot be diagnosed in the absence of any symptoms prior to age 12. When symptoms of what appears to be ADHD first occur after age 13, they are more likely to be explained by another mental disorder"

Diagnostic Categories for Uncertainty

1. Other Specified ADHD

* More structured approach to partial presentations Quote: "The other specified attention-deficit/hyperactivity disorder category is used in situations in which the clinician chooses to communicate the specific reason"

1. Unspecified ADHD

* For cases with insufficient information Quote: "includes presentations in which there is insufficient information to make a more specific diagnosis"

Key Evolution Points

1. Increasing Recognition of Complexity

* From relatively simple diagnostic uncertainty in DSM-III
* To more nuanced understanding of symptom presentation variability
* Greater acknowledgment of age and context effects

1. Diagnostic Categories

* Evolution from simple residual type
* To more sophisticated "Other Specified" and "Unspecified" categories
* Addition of severity specifiers

1. Assessment Approach

* Increased emphasis on multiple informants
* Greater recognition of recall reliability issues
* More detailed guidance on differential diagnosis

1. Cultural and Contextual Factors

* Growing recognition of cultural influences on diagnosis
* Increased attention to environmental contexts
* More sophisticated understanding of gender differences

Notable Changes Over Time

1. Language Precision
* More specific about what constitutes uncertainty
* Clearer guidelines for borderline cases
* Better defined categories for partial presentations
1. Clinical Guidance
* Increased detail about assessment challenges
* More specific recommendations for differential diagnosis
* Better defined thresholds for diagnosis
1. Flexibility in Classification
* More options for categorizing uncertain cases
* Better recognition of partial presentations
* More sophisticated approach to developmental variations

This analysis reveals a clear evolution in how diagnostic uncertainty has been addressed, with several key trends:
1. Increasing Sophistication: The manuals show a progression from relatively simple acknowledgment of diagnostic challenges to more nuanced understanding of how uncertainty manifests in different contexts and populations.
2. Better Structure: Later editions provide more structured ways to classify uncertain cases, moving from simple residual categories to more detailed "Other Specified" and "Unspecified" categories.
3. Cultural Awareness: There's growing recognition of how cultural and contextual factors contribute to diagnostic uncertainty.
One particularly interesting shift is in the language used to describe certainty levels - from relatively definitive statements in DSM-III to more nuanced acknowledgments of complexity in DSM-5 and 5-TR. Would you like me to elaborate on any particular aspect of this evolution?"

I'll analyze how diagnostic uncertainty is addressed across editions by examining language about diagnostic challenges, discussion of borderline cases, and evolution of 'Not Otherwise Specified' categories. Let me organize this systematically.

Analysis of Diagnostic Uncertainty in ADHD Across DSM Editions

DSM-III (1980)
Diagnostic Challenges

- Acknowledged fundamental uncertainty about disorder subtypes Quote: "It is not known whether they are two forms of a single disorder or represent two distinct disorders"

Borderline Cases

- Highlighted difficulty distinguishing from environmental factors Quote: "In such cases it may be impossible to determine whether the disorganized behavior is simply a

function of the chaotic environment or whether it is due to the child's psychopathology"

Residual Category

- Introduced "Residual Type" for partial presentations
- No explicit "Not Otherwise Specified" category

DSM-III-R (1987)
Diagnostic Challenges

- Added specificity about symptom variability Quote: "Signs of the disorder may be minimal or absent when the person is receiving frequent reinforcement or very strict control"

Borderline Cases

- More detailed discussion of differential diagnosis, especially with mood disorders
- Emphasized careful consideration in distinguishing from age-appropriate behavior

Diagnostic Uncertainty

- Required symptoms to be "considerably more frequent than that of most people of the same mental age"

DSM-IV (1994) & DSM-IV-TR (2000)
Diagnostic Challenges

- Expanded discussion of age-related diagnostic difficulties Quote: "It is especially difficult to establish this diagnosis in children younger than age 4 or 5 years, because their characteristic behavior is much more variable"

Diagnostic Uncertainty Language

- Introduced "In Partial Remission" category
- Added "Not Otherwise Specified" (NOS) category with specific examples

NOS Category Definition
Quote: "This category is for disorders with prominent symptoms of inattention or hyperactivity-impulsivity that do not meet criteria for Attention-Deficit/Hyperactivity Disorder"

DSM-5 (2013) & DSM-5-TR (2022)
Diagnostic Challenges

1. More explicit about recall reliability issues Quote: "Adult recall of childhood symptoms tends to be unreliable, and it is beneficial to obtain ancillary information"
2. Age-related uncertainty Quote: "ADHD cannot be diagnosed in the absence of any symptoms prior to age 12. When symptoms of what appears to be ADHD first occur after age 13, they are more likely to be explained by another mental disorder"

Diagnostic Categories for Uncertainty

1. Other Specified ADHD

- More structured approach to partial presentations Quote: "The other specified attention-deficit/hyperactivity disorder category is used in situations in which the clinician chooses to communicate the specific reason"

2. Unspecified ADHD

- For cases with insufficient information Quote: "includes presentations in which there is insufficient information to make a more specific diagnosis"

Key Evolution Points

1. Increasing Recognition of Complexity

- From relatively simple diagnostic uncertainty in DSM-III
- To more nuanced understanding of symptom presentation variability
- Greater acknowledgment of age and context effects

2. Diagnostic Categories

- Evolution from simple residual type
- To more sophisticated "Other Specified" and "Unspecified" categories
- Addition of severity specifiers

3. Assessment Approach

- Increased emphasis on multiple informants
- Greater recognition of recall reliability issues
- More detailed guidance on differential diagnosis

4. Cultural and Contextual Factors

- Growing recognition of cultural influences on diagnosis
- Increased attention to environmental contexts
- More sophisticated understanding of gender differences

Notable Changes Over Time

1. Language Precision

- More specific about what constitutes uncertainty
- Clearer guidelines for borderline cases
- Better defined categories for partial presentations

2. Clinical Guidance

- Increased detail about assessment challenges
- More specific recommendations for differential diagnosis
- Better defined thresholds for diagnosis

3. Flexibility in Classification

- More options for categorizing uncertain cases
- Better recognition of partial presentations
- More sophisticated approach to developmental variations

This analysis reveals a clear evolution in how diagnostic uncertainty has been addressed, with several key trends:

1. Increasing Sophistication: The manuals show a progression from relatively simple acknowledgment of diagnostic challenges to more nuanced understanding of how uncertainty manifests in different contexts and populations.
2. Better Structure: Later editions provide more structured ways to classify uncertain cases, moving from simple residual categories to more detailed "Other Specified" and "Unspecified" categories.
3. Cultural Awareness: There's growing recognition of how cultural and contextual factors contribute to diagnostic uncertainty.

One particularly interesting shift is in the language used to describe certainty levels - from relatively definitive statements in DSM-III to more nuanced acknowledgments of complexity in DSM-5 and 5-TR. Would you like me to elaborate on any particular aspect of this evolution?

YO Throughout this conversation, you have analyzed the complete DSM texts on ADD/ADHD, identified key differences across editions, and extracted relevant verbatim quotations. Now, using all the insights from this discussion, systematically organize your findings.

1. Summarize the most significant changes that occurred in each DSM edition.
2. Identify overarching trends in how ADHD has been defined, framed, and classified over time.
3. Analyze the implications of these changes for the evolving psychiatric discourse on ADHD.
4. Ensure your response is structured, referencing all prior analyses, and supporting key points with exact quotations from the DSM texts.

Based on our comprehensive analyses, here is a systematic organization of the key findings regarding ADHD's evolution across DSM editions:

1. SIGNIFICANT CHANGES BY EDITION

DSM-III (1980):

- Established "Attention Deficit Disorder" as primary name
- Created basic subtypes (with/without hyperactivity)
- Simple impairment criteria focused mainly on academic domains

- Quote: "Attention Deficit is the name given to this disorder, since attentional difficulties are prominent and virtually always present"

DSM-III-R (1987):

- Combined into single disorder "ADHD"
- Introduced formal severity levels
- Expanded environmental considerations
- Quote: "Signs of the disorder may be minimal or absent when the person is receiving frequent reinforcement or very strict control"

DSM-IV (1994) & DSM-IV-TR (2000):

- Introduced three subtypes (Combined, Predominantly Inattentive, Predominantly Hyperactive-Impulsive)
- Required impairment in multiple settings
- More comprehensive view of functional impact
- Quote: "There must be clear evidence of clinically significant impairment in social, academic, or occupational functioning"

DSM-5 (2013) & DSM-5-TR (2022):

- Changed age of onset criterion from 7 to 12 years
- Added severity specifiers
- Introduced cultural considerations
- Enhanced adult presentation descriptions
- Quote: "Clinical identification rates in the United States for African American and Latinx populations tend to be lower than for non-Latinx White populations"

2. OVERARCHING TRENDS

Diagnostic Framework Evolution:

- From simple categorical model to complex dimensional understanding
- Increased recognition of contextual factors
- Greater attention to developmental variations
- Growing emphasis on cultural competence

Assessment Approach:

- More structured diagnostic criteria
- Enhanced emphasis on multiple informants
- Better recognition of age-specific presentations
- Quote: "Adult recall of childhood symptoms tends to be unreliable, and it is beneficial to obtain ancillary information"

Gender and Cultural Considerations:

- More nuanced understanding of gender differences
- Growing recognition of cultural influences
- Increased attention to diagnostic disparities
- Quote: "Females are more likely than males to present primarily with inattentive features"

3. IMPLICATIONS FOR PSYCHIATRIC DISCOURSE

Conceptual Shifts:

- From childhood disorder to lifespan condition
- From primarily behavioral to neurodevelopmental framework
- From uniform to context-dependent presentation
- Quote: "The disorder is relatively stable through early adolescence, but some individuals have a worsened course"

Clinical Practice Evolution:

- More sophisticated assessment procedures
- Better recognition of comorbidities
- Enhanced cultural competence requirements
- Quote: "culturally competent diagnostic practices are relevant in assessing ADHD"

Research Implications:

- Growing emphasis on biological markers
- Recognition of genetic factors
- Increased attention to environmental influences
- Quote: "The heritability of ADHD is approximately 74%"

Social Impact:

- Better recognition of functional impairment across domains
- Enhanced understanding of long-term outcomes
- Greater attention to societal impact
- Quote: "Individuals with ADHD obtain less schooling, have poorer vocational achievement"

These changes reflect a broader evolution in psychiatric understanding, moving from:

1. Simple to complex models
2. Categorical to dimensional thinking
3. Uniform to culturally-informed approaches
4. Child-focused to lifespan perspectives

The evolution shows increasing sophistication in understanding ADHD as a complex, multifaceted disorder influenced by biological, psychological, and social factors. This has led to more nuanced diagnostic approaches and treatment considerations, while also highlighting the importance of cultural competence and contextual factors in assessment and treatment.

Would you like me to elaborate on any specific aspect of this evolution?

Copy Retry

Claude can make mistakes. Please double-check responses.
