# Supplementary material for: Four decades of ADHD: a systematic AI-assisted analysis of conceptual shifts across six DSM editions
Source: Front Psychiatry. 2026 Mar 9;17:1792051. doi: 10.3389/fpsyt.2026.1792051 (PMC13006570; doi:10.3389/fpsyt.2026.1792051)
Supplement: Supplementary file 3 [file DataSheet3.pdf]

Supplementary Material 3: Comparison of Agreed-Upon Key Differences

The following Prompt was entered to GPT-4o and Claude 3.5 Sonnet.

We examined the discourse on ADHD across six DSM editions using two distinct Large Language Models (GPT-4o and Claude 3.5 Sonnet). The LLMs generated insights on how ADHD has been described, framed, and classified over time.

Attached are two files:

1. **GPT-4o Analysis** – Contains the full responses generated by GPT-4o.
2. **Claude 3.5 Sonnet Analysis** – Contains the full responses generated by Claude 3.5 Sonnet.

Now, your task is to identify the key insights that both models independently recognized and agreed upon.

Use only the information from the attached LLM-generated analyses—do not introduce new interpretations or findings.

GPT-4o Response

Based on the analyses generated by GPT-4o and Claude 3.5 Sonnet, here are the key insights that both models independently recognized and agreed upon regarding the evolution of ADHD discourse across six DSM editions:

1. **Terminology and Classification Changes**

- Both models identified the shift from "Attention Deficit Disorder (ADD)" (DSM-III, 1980) to "Attention-Deficit/Hyperactivity Disorder (ADHD)" (DSM-III-R, 1987), with a consolidation of subtypes.
- DSM-IV (1994) introduced three subtypes, and DSM-5 (2013) relabeled them as "presentations" instead of fixed subtypes.
- This shift reflected an evolving understanding of ADHD as a more fluid and heterogeneous condition.

2. **Changes in Age of Onset**

- DSM-III to DSM-IV-TR (1980–2000) required symptoms to appear before age 7.
- DSM-5 (2013) & DSM-5-TR (2022) increased the onset threshold to age 12 to account for later-emerging symptoms and diagnostic challenges.
- This broadening of criteria made ADHD more inclusive and accounted for individual differences in developmental trajectories.

3. **Adjustments in Diagnostic Symptom Criteria**

- DSM-III (1980) required specific numbers of symptoms from separate categories (inattention, impulsivity, hyperactivity).

- DSM-IV (1994) introduced the six-symptom threshold for either inattention or hyperactivity-impulsivity.
- DSM-5 (2013) lowered the requirement for adults (≥17 years) from six to five symptoms, recognizing how ADHD manifests differently across the lifespan.

4. **ADHD as a Lifespan Condition**

- Both models emphasized that ADHD was initially seen as a childhood disorder (DSM-III, 1980), with limited discussion of persistence into adulthood.
- By DSM-5 (2013), ADHD was fully recognized as a condition that persists into adulthood, requiring separate criteria for older adolescents and adults.
- This reflects a major shift in discourse from ADHD being "outgrown" to being a lifelong neurodevelopmental disorder.

5. **Increasing Recognition of Functional Impairment**

- DSM-III (1980) primarily described ADHD as a set of symptoms, while later editions focused on functional impairment in school, work, and social settings.
- DSM-IV (1994) and later editions required impairment in at least two settings (home, school, work, social life).
- DSM-5-TR (2022) emphasized the impact of ADHD on development, making impairment a core diagnostic consideration.

6. **Expanded Understanding of Comorbidities**

- Early DSM editions (DSM-III to DSM-IV) primarily associated ADHD with conduct disorder and learning disabilities.
- Later editions (DSM-5 and DSM-5-TR) recognized extensive comorbidities, including autism spectrum disorder, mood disorders, anxiety, substance use disorders, and suicidal risk.
- This shift reflects an evolving understanding of ADHD as part of a broader neurodevelopmental and psychiatric landscape.

7. **Growing Consideration of Cultural and Gender Bias**

- DSM-III to DSM-IV-TR (1980–2000) did not address cultural or gender disparities.
- DSM-5-TR (2022) explicitly acknowledged diagnostic biases, including underdiagnosis in females and racial/ethnic minorities.
- Both models highlighted the addition of clinician bias discussions in DSM-5-TR, signaling a broader shift in psychiatry toward addressing disparities in mental health diagnosis.

8. **From a Behavioral Disorder to a Neurodevelopmental Condition**

- DSM-III (1980) framed ADHD primarily as a disorder of hyperactivity and poor impulse control.

- DSM-5 (2013) and DSM-5-TR (2022) emphasized executive dysfunction, cognitive regulation, and genetic heritability (~74%).
- Both models noted the increasing medicalization and biological framing of ADHD, with later editions integrating brain volume differences, neuroimaging findings, and genetic markers.

9. **Introduction of Severity Specifiers**

- DSM-5 (2013) introduced severity levels (Mild, Moderate, Severe).
- Both models identified this as a shift toward a more dimensional approach to ADHD, allowing for greater diagnostic flexibility.

10. **Expanding Contextual Understanding**

- DSM-III (1980) focused mainly on school and home environments.
- Later editions expanded the discussion to include workplace challenges, relationships, and broader social contexts.
- DSM-5-TR (2022) acknowledged symptom variability across settings, including digital environments (e.g., electronic screens).

11. **Evolving Debate on Overdiagnosis**

- DSM-III (1980) estimated ADHD prevalence at ~3%, whereas DSM-5-TR (2022) estimates it at ~7.2%.
- Both models recognized that this increase in prevalence reflects both better recognition and concerns about overdiagnosis.
- This debate aligns with broader concerns in psychiatry about medicalization and diagnostic expansion.

Conclusion: Shared Key Insights

The consensus between GPT-4o and Claude 3.5 Sonnet highlights:

1. The increasing complexity and inclusivity of ADHD diagnosis over time.
2. A shift from a childhood behavioral disorder to a lifelong neurodevelopmental condition.
3. Growing awareness of comorbidities, cultural biases, and gender differences.
4. A shift from rigid subtypes to fluid presentations, reflecting a dimensional approach to diagnosis.
5. The expansion of ADHD from hyperactivity to a broader framework including executive dysfunction and cognitive regulation.

These findings indicate that psychiatric discourse on ADHD has evolved in response to new research, societal changes, and a push for more comprehensive and inclusive diagnostic criteria.

Would you like a visual summary (e.g., a comparative table or chart) to illustrate these points clearly? 🚀

YO: yes.

Evolution of ADHD in DSM Editions

**Key Aspect**	**DSM-III (1980)**	**DSM-III-R (1987)**	**DSM-IV (1994)**	**DSM-IV-TR (2000)**	**DSM-5 (2013)**	**DSM-5-TR (2022)**
Terminology & Classification	ADD with/without Hyperactivity	ADHD (subtypes removed)	ADHD with three subtypes	Same as DSM-IV	ADHD 'presentations' replace subtypes	Same as DSM-5
Age of Onset	Symptoms must appear before age 7	Symptoms must appear before age 7	Symptoms must appear before age 7	Same as DSM-IV	Onset threshold raised to age 12	Same as DSM-5
Diagnostic Criteria	Three symptoms each from inattention, impulsivity, hyperactivity	Single list of 14 symptoms (8 required for diagnosis)	Six symptoms in either inattention or hyperactivity-impulsivity	Minor refinements, further clarified criteria	Adult criteria adjusted (≥5 symptoms vs. 6 for children)	Further refinement, emphasizing contextual variability
ADHD as a Lifespan Condition	Primarily a childhood disorder	Some recognition of persistence into adolescence	Recognition of adolescent ADHD	More explicit mention of adult ADHD but not yet a focus	Fully recognized as a lifespan disorder	Continued emphasis on adult ADHD
Functional Impairment	Behavioral symptoms emphasized	Still focused on behavior, but with broader symptom inclusion	Impairment required in at least two settings	Further clarification on impairment in multiple settings	Functional impairment emphasized	Clarified impact on development
Comorbidities	Linked mainly to conduct disorder & learning disabilities	Greater acknowledgment of ODD and conduct disorder	More detailed comorbidities (ODD, anxiety, mood disorders)	More discussion of learning disabilities & psychiatric comorbidities	Expanded comorbidities (ASD, SUD, PTSD, suicidality)	More focus on gender-specific comorbidities
Cultural & Gender Bias	No discussion of gender or cultural biases	No explicit gender or cultural focus	Basic mention of gender disparities (higher in boys)	Some emerging acknowledgment of cultural differences	Acknowledgment of diagnostic disparities	Explicit discussion of racial/ethnic biases

Neurodevelopmental Framing	Hyperactivity-focused, minimal neurobiological discussion	More mention of neurological basis but still limited	Increased focus on executive functioning	Still framed largely as behavioral, but cognitive elements increasing	Genetic heritability, brain volume differences noted	Stronger emphasis on neurobiological underpinnings
Severity Specifiers	No severity levels specified	No severity levels specified	Still no formal severity specifiers	Still no formal severity levels	Introduction of Mild, Moderate, Severe levels	Severity levels reinforced
Contextual Understanding	School & home contexts only	More discussion of symptom manifestation variability	More detail on symptom variation across settings	Some mention of symptom fluctuation based on context	Recognition of digital and work environments	Expanded analysis of sociocultural factors
Prevalence & Overdiagnosis	~3% prevalence, seen as a rare disorder	Diagnosis becoming more common, but not widely debated yet	Recognition of increasing diagnoses but no major concern yet	Some early concerns about diagnostic inflation	~7.2% prevalence, concerns about overdiagnosis emerge	Increased awareness of diagnostic inflation concerns

Claude 3.5 Sonnet Response

Based on analyzing the outputs from both GPT-4o and Claude 3.5 Sonnet, here are the key insights about ADHD's evolution in the DSM that both models independently identified and agreed upon:

1. **Evolution from Behavioral to Neurodevelopmental Framework**

- Both models highlighted how ADHD shifted from being viewed primarily as a behavioral disorder in DSM-III to being recognized as a complex neurodevelopmental condition in later editions.

- They noted the progression from focusing on observable behaviors to incorporating neurobiological and genetic understanding.

2. **Expansion from Childhood to Lifespan Disorder**

- Both models emphasized the significant shift from viewing ADHD as exclusively a childhood condition to recognizing it as a disorder that can persist throughout life.

- They identified DSM-5's formal recognition of adult ADHD as a crucial development, including specific diagnostic adaptations for adults.

3. **Increased Recognition of Contextual Factors**

- Both analyses highlighted how later DSM editions increasingly acknowledged that ADHD symptoms vary across different settings and contexts.

- They noted the growing emphasis on how environmental factors influence symptom expression.

4. Evolution of Gender Understanding

- Both models traced the progression from viewing ADHD as predominantly affecting males (10:1 ratio in DSM-III) to recognizing different presentation patterns in females.

- They highlighted the DSM-5-TR's acknowledgment of gender-specific manifestations, particularly the tendency for females to show more inattentive symptoms.

5. Growing Cultural Awareness

- Both analyses emphasized the DSM's increasing recognition of cultural factors in diagnosis, particularly in DSM-5 and DSM-5-TR.

- They noted the explicit acknowledgment of diagnostic disparities affecting minority populations in later editions.

6. Changes in Diagnostic Criteria

- Both models identified key changes in diagnostic requirements across editions, including:

  - The shift in age of onset from 7 (DSM-III through DSM-IV-TR) to 12 years (DSM-5)

  - The evolution from rigid subtypes to more flexible "presentations"

  - The introduction of severity specifiers

7. Expanding Concept of Impairment

- Both analyses traced how the understanding of functional impairment evolved from primarily academic difficulties to a comprehensive view encompassing social, occupational, and personal domains.

- They noted the increasing emphasis on documenting impairment across multiple settings.

8. Recognition of Diagnostic Complexity

- Both models highlighted the progression from simple, categorical diagnostic approaches to more nuanced, dimensional understanding.

- They emphasized the growing recognition of symptom variability and the need for comprehensive assessment approaches.

This analysis is based strictly on the overlapping insights identified independently by both LLMs in their examination of the DSM texts, representing areas of strong consensus in their interpretations.
