# Supplementary material for: Four decades of ADHD: a systematic AI-assisted analysis of conceptual shifts across six DSM editions
Source: Front Psychiatry. 2026 Mar 9;17:1792051. doi: 10.3389/fpsyt.2026.1792051 (PMC13006570; doi:10.3389/fpsyt.2026.1792051)
Supplement: Supplementary file 4 [file DataSheet4.pdf]

Supplementary Material 4: Final Structural Comparison of the Agreed-Upon Differences

A final structured comparison of the agreed-upon differences between the various editions of the DSM was performed by GPT-4o using the following prompt:

"Attached is a Word file containing the responses of both models (GPT-4o and Claude 3.5 Sonnet) to the task of identifying key insights that both independently recognized and agreed upon. GPT-4o's response appears first, followed by Claude 3.5 Sonnet's response.
Please compare the two responses systematically, relying only on the attached file. Specifically:

Identify the key insights that both models independently recognized.

Highlight any differences or nuances in how each model articulated these insights.

Provide a structured summary that organizes these findings clearly. Do not incorporate external knowledge—base your analysis strictly on the content of the attached file."

GPT-4o Response

Below is a structured summary comparing the responses from GPT-4o and Claude 3.5 Sonnet, strictly based on the provided document. The comparison includes key insights both models independently recognized and differences in articulation or emphasis.

1. Key Insights Both Models Independently Recognized

Key Insight	Shared Understanding by Both Models
Shift from Behavioral to Neurodevelopmental Framework	Initially viewed as a behavioral disorder (DSM-III) but later recognized as a neurodevelopmental condition (DSM-5, DSM-5-TR), incorporating genetic, neurobiological, and cognitive components.
Expansion from Childhood to Lifespan Disorder	ADHD was originally seen as a disorder of childhood, but later editions (DSM-5) formally recognized that ADHD persists into adulthood, requiring different diagnostic thresholds for adults.
Changes in Age of Onset	The diagnostic threshold changed from symptoms before age 7 (DSM-III to DSM-IV-TR) to before age 12 (DSM-5) to accommodate cases with later-emerging symptoms.
Diagnostic Criteria Evolution	ADHD's classification evolved from rigid subtypes (DSM-III, DSM-IV) to more flexible presentations (DSM-5). Additionally, DSM-5 introduced severity specifiers (Mild, Moderate, Severe).

Recognition of Functional Impairment	Earlier editions focused on ADHD symptoms, while later editions emphasized functional impairment in academic, social, and occupational settings.
Growing Recognition of Contextual Factors	The role of environmental influences on ADHD symptoms became more prominent in later DSM editions, reflecting an ecological perspective on symptom expression (e.g., digital environments, workplace settings).
Expanded Understanding of Comorbidities	Early DSM editions linked ADHD mostly with conduct disorder and learning disabilities, whereas later editions (DSM-5, DSM-5-TR) acknowledged comorbidities with autism spectrum disorder, mood disorders, substance use disorders, and anxiety disorders.
Increasing Awareness of Gender Differences	Early DSM editions emphasized ADHD as a male-dominant condition (DSM-III: 10:1 male-to-female ratio). Later editions (DSM-5-TR) recognized different presentation patterns in females (e.g., more inattentive symptoms).
Growing Cultural Awareness	Earlier DSM editions lacked cultural considerations. DSM-5-TR explicitly acknowledged racial and ethnic disparities, including diagnostic bias and underdiagnosis in minority populations.
Expanding Concept of Impairment	Functional impairment broadened beyond school performance to include work, relationships, and self-regulation, requiring documentation across multiple settings.
Recognition of Diagnostic Complexity	Earlier editions used strict categorical classifications, while later editions moved toward a dimensional approach, acknowledging symptom variability and partial remissions.
Debate on Overdiagnosis	ADHD prevalence increased from ~3% (DSM-III) to ~7.2% (DSM-5-TR), raising concerns about overdiagnosis and medicalization.

2. Summary of Key Differences

Aspect	GPT-4o's Focus	Claude 3.5 Sonnet's Focus
Scientific & Medical Framing	Strong emphasis on biological and genetic underpinnings of ADHD.	Emphasizes cognitive and executive functioning changes over time.

Functional Impairment	Highlights occupational, academic, and social impairments.	Focuses more on contextual variation and environmental adjustments.
Cultural and Gender Bias	Discusses specific examples of underdiagnosis and diagnostic bias.	Frames the issue within social justice and equity concerns.
Dimensional vs. Categorical Diagnosis	Acknowledges movement toward a spectrum-based understanding of ADHD.	Emphasizes the shift in diagnostic philosophy from rigid categories to nuanced clinical assessment.

3. Final Takeaways

1. Both models recognized the same broad trends in the evolution of ADHD diagnosis, with strong agreement on key milestones (e.g., changing age of onset, shift from subtypes to presentations, recognition of adult ADHD, increased cultural awareness, expansion of functional impairment).
2. GPT-4o focused more on biological, neurodevelopmental, and medical aspects, while Claude 3.5 Sonnet leaned toward cognitive, contextual, and social dimensions.
3. Claude placed more emphasis on gender and cultural disparities, while GPT-4o provided more granular details on biological findings and diagnostic thresholds.

This comparison highlights how both models provide complementary perspectives on the same dataset, reinforcing the robustness of their insights.
